# Shotgun Metagenomic Profiling of the Gut Virome in Prodromal and Confirmed Parkinson's Disease

**DOI:** 10.1002/ana.78243

**Published:** 2026-05-27

**Authors:** Deepika Dinesh, Xochitl C. Morgan, Jordan Jensen, Kjetil Bjornevik, Michael A. Schwarzschild, Alberto Ascherio, Curtis Huttenhower, Natalia Palacios

**Affiliations:** ^1^ Department of Public Health University of Massachusetts Lowell Lowell MA; ^2^ Department of Biostatistics Harvard T. H. Chan School of Public Health Boston MA; ^3^ Harvard Chan Microbiome in Public Health Center (HCMPH) Harvard T. H. Chan School of Public Health Boston MA; ^4^ Department of Immunology and Infectious Diseases Harvard T. H. Chan School of Public Health Boston MA; ^5^ Department of Nutrition Harvard T. H. Chan School of Public Health Boston MA; ^6^ Channing Division of Network Medicine, Department of Medicine Brigham and Women's Hospital and Harvard Medical School Boston MA; ^7^ Department of Neurology Massachusetts General Hospital Boston MA; ^8^ Broad Institute of MIT and Harvard Cambridge MA; ^9^ Department of Veterans Affairs ENRM VA Hospital Bedford MA

## Abstract

We conducted a nested case‐control study within the Nurses’ Health Study and the Health Professionals Follow‐up Study to examine the role of the gut virome (GV) in Parkinson's disease (PD). We applied a novel metagenomic virome profiling approach, Bioinformatic Application for Quantification and Labeling of Viral taxonomy (BAQLaVa), to prospectively collected metagenomic data from 62 participants with PD, 123 healthy controls, and 90 participants with prodromal PD (pPD). Multivariate linear modeling identified 3 viral genome bins (VGBs) that were elevated in PD: MVG081219 (*β* = 0.86, *q* = 0.013), MVG041501 (*β* = 0.95, *q* = 0.048), MVG081211 (*β* = 0.66, *q* = 0.048) and one VGB, MVG098915 (*β* = −1.42, *q* = 0.047) that was depleted in participants with PD compared to controls. These four VGBs were similarly associated with pPD. This work suggests that the GV has potential as a future biomarker for PD. ANN NEUROL 2026;100:334–340

Parkinson's disease (PD) is a progressive neurodegenerative disorder caused by the degeneration of dopaminergic neurons in the substantia nigra, characterized by motor symptoms, including tremor, rigidity, bradykinesia, and postural instability. Past studies, including ours,^1^ have implicated gut microbial dysbiosis in PD. Endogenous viruses may also impact PD risk^2^ due to their role in immune activation and subsequent phosphorylation and aggregation of alpha‐synuclein.^3^ Although this has been previously studied in the context of pathogenic eukaryotic viruses, endogenous viruses in the human gut are diverse, overwhelmingly phage (>97%), and interact with the host and surrounding microbial community in a variety of ways.

Alterations in the gut virome (GV) can impact the composition and function of gut bacteria, potentially enabling horizontal gene transfer,^4^, ^5^ supplying genes key to carbohydrate or toxin metabolism, and modulating bacterial growth and fitness.^6^, ^7^ The GV can also, in some instances, be a source of antibiotic resistance and virulence factors^8^ and contribute to non‐host derived immunity.^9^ The GV thus represents an underexplored layer in microbiota–gut–brain communication. The association between the endogenous GV and PD is under‐studied, with early reports suggesting lower gut viral abundance and increase in lytic lactococcal phages in PD.^10^

Here, we examined the GV in PD and prodromal PD (pPD) in 2 large cohorts, the Health Professionals Follow‐up Study [HPFS] and the Nurses’ Health Study [NHS], applying a recently developed viral profiling method, Bioinformatic Application for Quantification and Labeling of Viral taxonomy (BAQLaVa), which is appropriate for unenriched shotgun metagenomic sequencing data. Most previous methods have focused on either identification of putatively viral contigs after assembly (eg, VirFinder^11^ among many others) or on sequences generated from experimental enrichments for virus‐like particles (VLPs).^12^ BAQLaVa sensitively and specifically quantifies viral reads assigned taxonomy from unenriched shotgun metagenomes, building on methodology developed for other types of microbial taxonomic profiling in the bioBakery.

## Methods

### Institutional Review Board Approval

The study protocol was approved by the institutional review boards of the Brigham and Women's Hospital and the Harvard T. H. Chan School of Public Health.

### PD Phenotypes

All study participants were members of the NHS or the HPFS, described previously.^1^ Details for classification of participants as PD, pPD, and healthy controls, and methods for fecal sample collection and sequencing were outlined in prior work.^1^ Briefly, ascertainment of PD was based on self‐report, followed by neurologist confirmation (author M.S.). Healthy controls were selected among NHS and HPFS participants who did not self‐report PD on the bi‐annual questionnaire and did not have any symptoms of pPD, such as constipation, smell loss, or probable rapid eye movement (REM) Sleep Behavior Disorder (pRBD). The patients with pPD were selected based on a combination of (a) self‐reported constipation, (b) self‐reported pRBD, and (c) substantial smell loss ascertained as a score of 7 or below on the Brief Smell Identification Test,^1^ which, in a case‐control study in our cohort associated with an odds ratio (OR) of 160 (95% confidence interval [95% CI] = 72.8 to 353, *p* < 0.0001) of PD.^13^

Included in this study were 62 participants with PD (diagnosis in 2006 or later), 123 healthy controls, and 90 participants with pPD who had stool samples collected between December 12, 2018, and January 18, 2022. All participants were invited to provide 2 sets of stool samples, an average of 24.6 months apart (range = 5.6–37.6 months between collection) (Table1).

**TABLE 1:** Characteristics of Nurses’ Health Study and Health Professionals Follow‐Up Study Participants With GV profiling.

	Healthy Controls	Prodromal PD	PD
Total stool collection, N	123	90	62
Returned first collection sample	113	81	57
Date (mean) of first sample^a^	May 2019	May 2019	Aug 2019
Date (range) of first sample^a^	Dec 2018 to Jan 2020	Dec 2018 to Jan 2020	Dec 2018 to Jun 2020
Participant characteristics			
Age, yr	80.7 (5.4)	80.2 (5.6)	79.3 (5.0)
Sex, F (%)	43.1	40.0	54.8
Body mass index, kg/m^2^	26.3 (5.0)	26.2 (3.9)	25.4 (5.4)
Med Diet Score^b^	4.6 (2.1)	3.9 (2.0)	4.4 (2.3)
Alcohol intake, g/day	10.4 (11.6)	11.5 (14.8)	7.2 (8.9)
Caffeine intake, g/day	157.7 (117.3)	156.9 (124.3)	129.1 (94.9)
Pack yr smoking	7.8 (14.0)	9.3 (15.4)	NA (NA)
Year first PD symptoms, mean^a^			May 2010
Year first PD symptoms, range			Sep 2004 to Jun 2015
Year PD diagnosis, mean^a^			Nov 2011
Year PD diagnosis, range			Dec 2005 to Jun 2015
Bristol stool scale			
1–2, hard stool	10.6%	22.2%	33.9%
3–5, normal stool	74.8%	64.4%	54.8%
6–7, loose stool	13.8%	12.2%	6.5%
Returned second collection sample	73	46	26
Repeat sample	63	37	21
Collection 2 only sample	10	9	5
Date (mean) of second sample^a^	Apr 2021	Apr 2021	Sep 2021
Date (range) of second sample^a^	Dec 2020 to Nov 2021	Dec 2020 to Jan 2022	Aug 2021 to Oct 2021

*Note:* Characteristics of all participants who participated in the study, characteristics are reported at the time of first collection for all participants.

^a^ Collection, diagnosis and symptom onset mean date computed as arithmetic mean of non‐missing dates, rounded to the nearest day and shown.

^b^ Mediterranean Diet adherence was assessed via Food Frequency Questionnaire and scored according to Trichopoulou et al.^21^

GV = gut virome; Med diet = Mediterranean diet; NA = not applicable; PD = Parkinson's disease.

Participants who reported a colonoscopy, hospital stay, and injected or oral antibiotic use within 2 months prior to stool collection were not eligible to give a sample in that collection round.

### Bioinformatic Application for Quantification and Labeling of Viral Taxonomy

After quality control and host read removal via the KneadData version 0.3 pipeline, viral profiling was performed using the BAQLaVa V0.1 (https://huttenhower.sph.harvard.edu/baqlava), which assigns viral and bacteriophage viral genome bins (VGBs) from shotgun DNA sequencing data. Briefly, BAQLaVa uses 2 databases (nucleotide and protein) in a 2‐tier reference‐based mapping approach inspired by HUMAnN.^16^ The nucleotide database comprises de‐replicated genomes from viral RefSeq, and viral Metagenome‐Assembled Genomes (vMAGs) from the Gut Virome Database^14^ and the Viral Sequence Clusters database. The protein database comprises UniRef90s^15^ that map to viral exemplar genomes from the International Committee on Taxonomy of Viruses (ICTV). Sequencing reads not mapping to genomes in the nucleotide search step continue to the translated search. BAQLaVa considers nucleotide features “present” when >=50% of the genome is covered. For protein features, >=50% of an ICTV species’ defined Uniref90 protein set must be present, with each individual Uniref90 >=50% coverage. For VGBs composed of vMAGs that were not annotated to a viral species, we assigned putative hosts to the genomes using the integrated Phage‐Host Prediction tool (iPHoP) version 1.3.2, requiring a minimum score of 90^17^ (database: Aug_2023_pub_rw). We applied an abundance and prevalence filter for VGBs, requiring a relative abundance >10^−5^ (0.01%) in at least 10% of samples and conducted sensitivity analyses without filtering. For VGBs associated with PD at false discovery rate (FDR) *q* < 0.05, VGB sequences were retrieved from the BAQLaVa (version 0.1) database, and Megablast (using the nr database and default parameters) was used to identify the best hit for each VGB sequence. The alignment and corresponding GenBank file were exported for each best hit and used for visualization.

### Statistical Analysis

#### Overall Community Patterns of Microbial Variation

The Bray–Curtis dissimilarity metric was used for beta‐diversity analyses. We used ordination via Principal Coordinates Analyses (PCoA) to visualize beta‐diversity relationships and repeated permutational multivariate analysis of variance (PERMANOVA) of Bray–Curtis dissimilarities to quantify the percent variance explained by phenotype group (PD/pPD/healthy control). Analogously, we used the Shannon index for alpha diversity analyses, in the context of a linear mixed model (LMM), treating subject as a random effect.

#### Feature‐Wise Analyses

The feature‐wise analyses were performed using MaAsLin 2,^18^ under default parameters. The Benjamini–Hochberg FDR was used to control type I error with *q* < 0.05 considered as statistically significant. Models were adjusted for batch, age (continuous), sex (categorical), adherence to Mediterranean Dietary pattern (MED diet, continuous), pack years of smoking (continuous), body mass index (BMI, continuous), Bristol Stool Scale (continuous), and date (in days, continuous, mean‐centered) of stool collection. Participant ID was included in the model as a random effect, to account for repeat sampling.

#### Covariates

The PERMANOVA beta diversity, Shannon alpha diversity, and MaAsLin 2 feature‐wise models were adjusted for age, sex, adherence to the MED diet, pack years of smoking, BMI, Bristol Stool Scale, and date of stool collection.

#### Random Forest Classifier

Models were fit using the *scikit‐learn* package in Python software, using default parameters, and included cross‐validation based on 100 random splits in an 80/20 ratio, with repeat samples from the same subject placed together in training and testing folds (Python *ShuffleSplit*). The receiver operating characteristic area under the curve (ROC AUC) over 100 random forest (RF) classifier iterations were used to assess RF classifier performance. The RF was fit to both confirm feature‐wise analyses from MaAsLin 2 and to estimate extent of population‐level shift in GV composition associated with PD.

## Results

**FIGURE 1:**
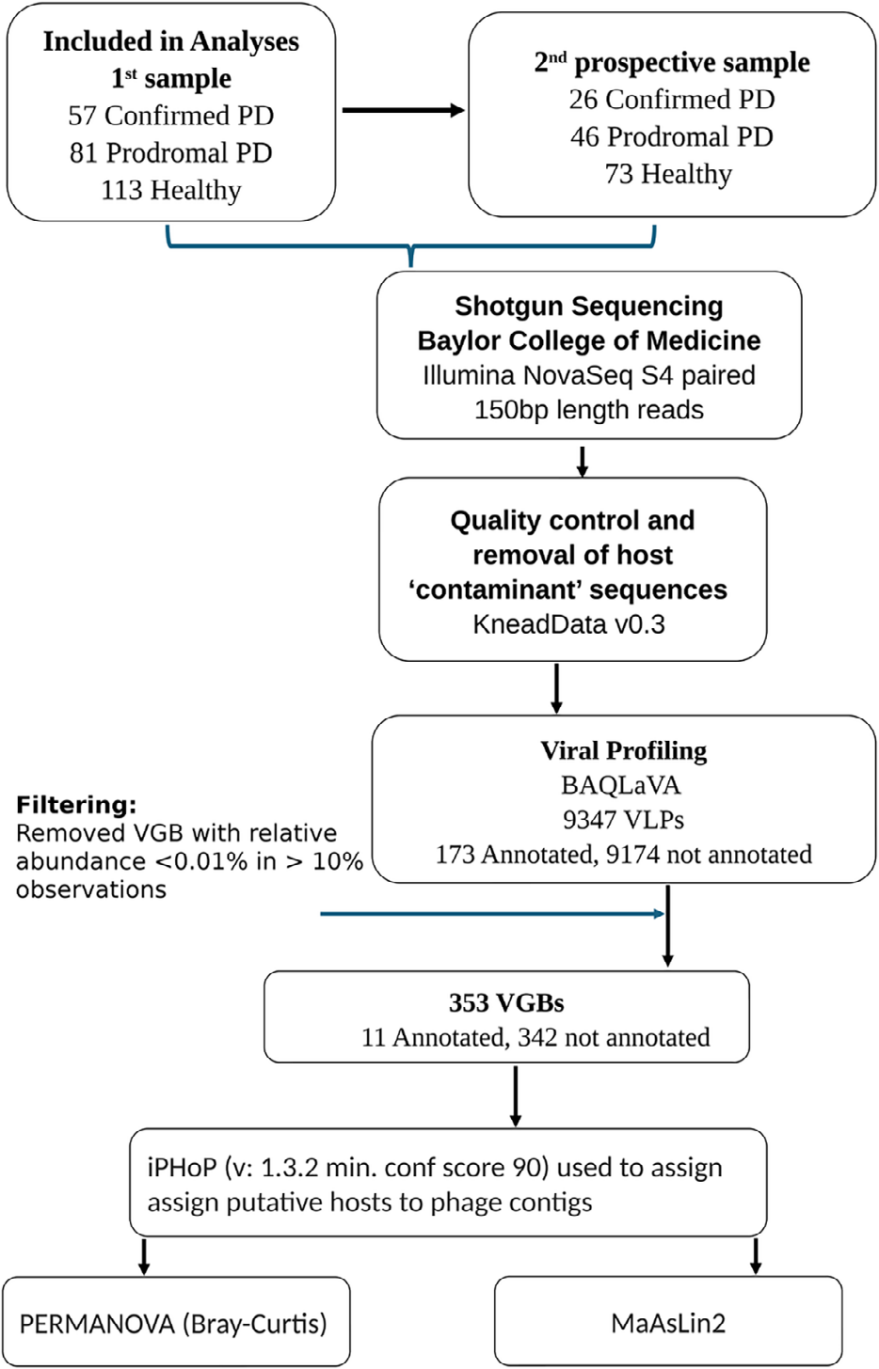
STORMS Chart summarizing gut virome data generation and analysis. [Color figure can be viewed at www.annalsofneurology.org]

The STORMS outline for the study is presented in Figure 1, and baseline characteristics of our study participants are shown in the Table 1. None of the study participants changed disease status from healthy to pPD or from pPD to PD between the 2 collections. BAQLaVa identified 9,347 unique VGBs among 396 total metagenomes from the 275 participants in our dataset, of which 173 (1.8%) were taxonomically identified. The remainder (9,174, 98.2%) were taxonomically unclassified at the genus level, consistent with the large amount of viral “dark matter” in the human gut.^19^

### Gut Viral Ecology Differentiates Participants With PD From Controls

**FIGURE 2:**
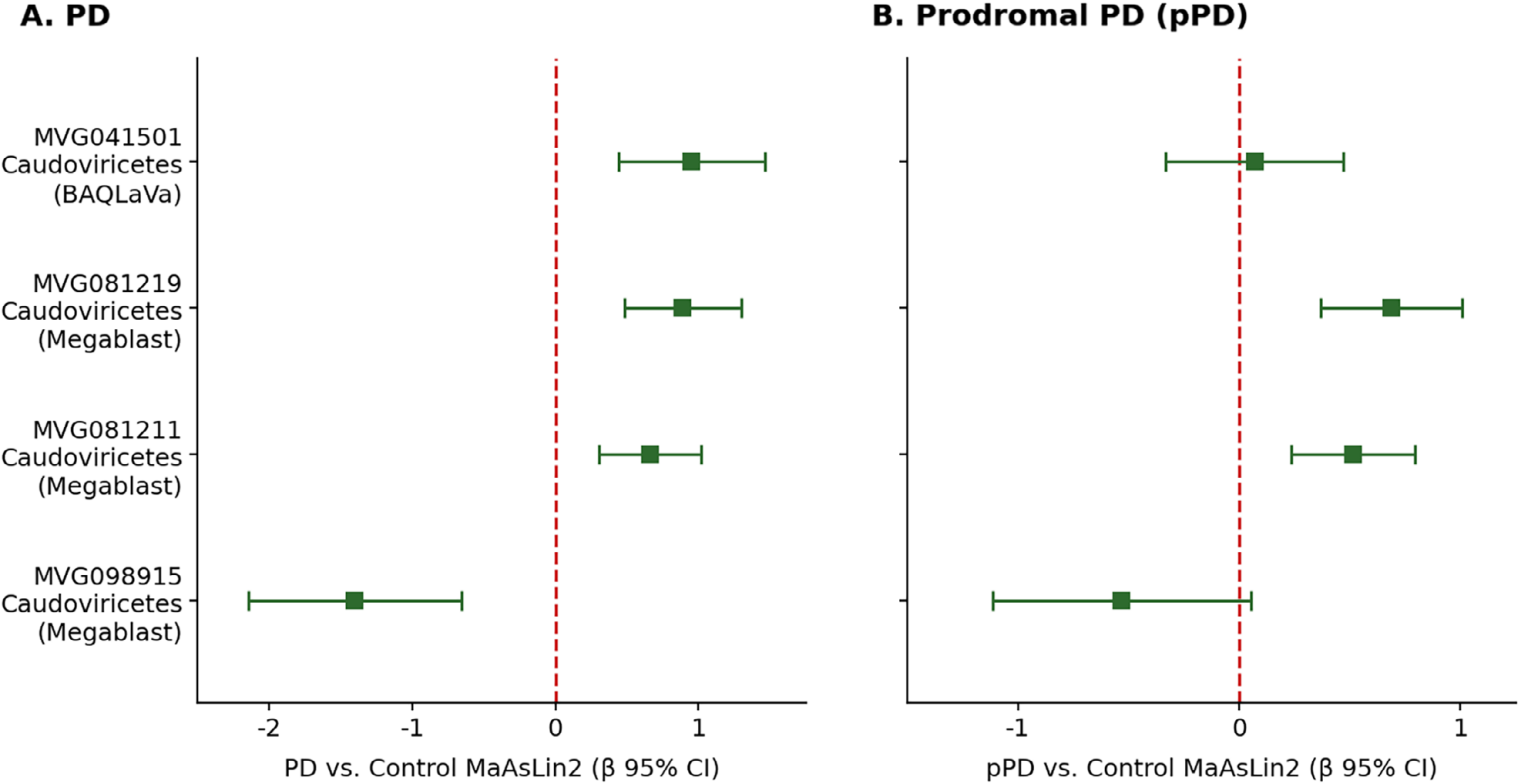
VGBs associated with PD and pPD in the NHS and HPFS. VGBs associated with (A) PD and (B) prodromal PD a multivariate MaAsLin2 model adjusted for subject as random effect and sequencing batch, age, sex, adherence to MED diet, pack years of smoking, BMI, and Bristol scale as fixed effects. Plotted are VGBs significantly (*q* < 0.05) associated with PD and pPD after FDR correction. MaAsLin2 beta coefficients are from multivariate models fitted to the log_2_ relative abundances of VGBs, comparing PD and pPD to healthy controls. [Color figure can be viewed at www.annalsofneurology.org]

Phenotype (PD/pPD/healthy controls) explained a small but statistically significant proportion of variance in viral community structure (PERMANOVA *R* ^2^ = 0.009, *p* = 0.03). MaAsLin2 identified 3 VGBs elevated, and 1 VGB depleted, in PD (Fig 2), all *Caudoviricetes*. These included MVG081219 (*βMaAsLin2* = 0.86, *q* = 0.013); MVG041501 (*βMaAsLin2* = 0.95, *q* = 0.048); and MVG081211 (*βMaAsLin2* = 0.66, *q* = 0.048). One VGB, MVG098915 (*βMaAsLin2* = −1.42, *q* = 0.047) abundance was reduced in PD compared with healthy controls. One VGB, MVG098915 (*βMaAsLin2* = −1.34, *q* = 0.04) had reduced abundance in PD compared with healthy controls. All 4 VGBs significantly associated with PD phenotypes, were identified as *Caudoviricites* in BAQLaVa as well as via Megablast. The aligned regions showed many of the essential regions of tailed dsDNA phages, including capsid assembly (capsid and portal proteins), tail assembly, DNA packaging (terminase), and lysis (holin), as well as multiple putative transcription factors (Supplementary Table S1).

Three VGBs with elevated abundance in PD also had higher abundance in pPD: MVG081219 (*βMaAsLin2* = 0.69, *q* = 0.01) and MVG041501 (*βMaAsLin2*_=_ 0.52, *q* = 0.04). For the other 3 VGBs significant in PD, although not significant in pPD, the associations were in the same direction (see Fig 2).

**FIGURE 3:**
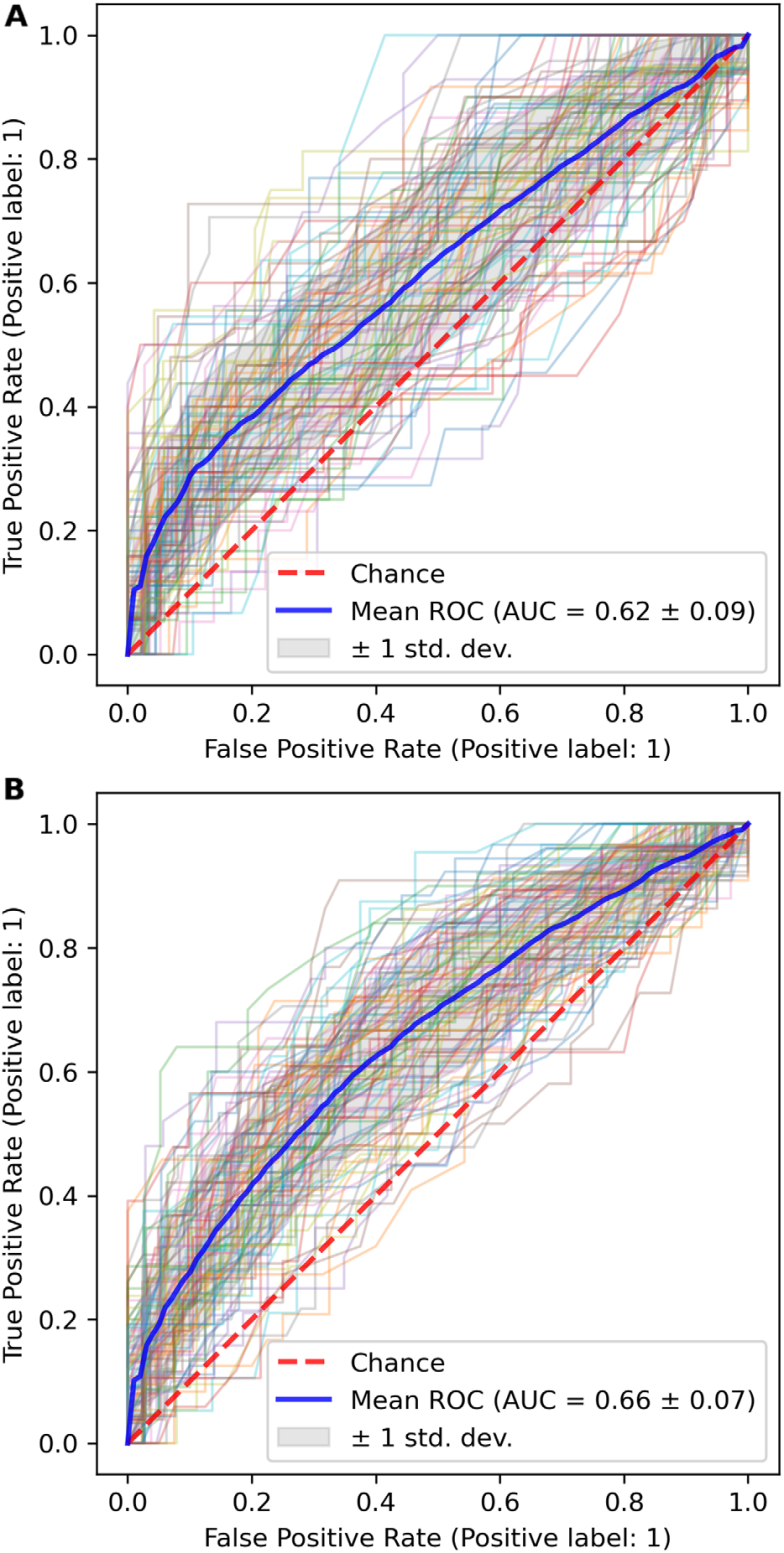
Overall gut viral community structure differentiates (A) PD and (B) pPD from healthy controls. The ROC performance of a RF classifier based on discriminating between (A) PD and (B) pPD from healthy controls using metagenomically derived viral taxonomic profiles. PD = Parkinson’ disease; pPD = prodromal Parkinson's disease; RF = random forest; ROC = receiver operating characteristic. [Color figure can be viewed at www.annalsofneurology.org]

A RF classifier had modest but significant accuracy (AUC = 0.62) to discriminate between PD and healthy controls based on all profiled VGBs (Fig 3A). Three of the VGBs significantly associated with PD in MaAsLin2 analyses (MVG081219, MVG041501, and MVG098915) were among the top 20 VGBs with highest feature importance in the RF model. For pPD, the RF had an AUC of 0.66 and MVG081219, MVG041501, and MVG081211 and were again among the VGBs with highest importance (Fig 3B).

## Discussion

To our knowledge, this is the first study using metagenomic GV profiling to study PD and pPD. We identified 3 VGBs with elevated, and 1 VGB with reduced, abundances in participants with PD compared with healthy controls, with similar associations in pPD. These results are overall consistent with our prior publication on gut bacteria in this cohort^1^ and suggest a parallel shift in the virome in prodromal and clinically diagnosed PD.

The consistent direction of virome alterations in both pPD and PD observed here suggests a potential association between the GV and PD etiology. This finding, however, is exploratory and requires confirmation in larger studies. The 4 viral VGBs associated with PD in our analyses were classified within the class *Caudoviricetes*, tailed double‐stranded DNA bacteriophages, by either BAQLaVA or MegaBlast. *Caudoviricetes* are widely recognized as the dominant component of the human GV across both healthy individuals and diverse disease states.^20^ This class encompasses a highly diverse group of phages that infect many of the major bacterial taxa in the gut microbiome. In the context of PD, *Caudoviricetes* phages may contribute to shaping microbial community structure through lytic and lysogenic interactions with bacterial hosts, thereby influencing gut dysbiosis and potentially modulating gut–brain axis signaling. Despite their ubiquity and apparent ecological importance, the functional consequences of *Caudoviricetes* variation in the gut remain incompletely understood. Our findings highlight the importance of further investigating *Caudoviricetes*–bacterial interactions in PD and underscore the need for deeper functional characterization of this dominant and diverse viral class.

Our study was nested within carefully designed prospective cohorts, which reduces the possibility of selection bias. This is also the first PD GV study to date with a carefully defined prodromal group, in addition to a group of participants with clinician characterized and confirmed PD. Including a prodromal group allowed us to examine whether prodromal changes in the GV resemble those in PD, as was the case in our prior work on the bacteriome.^1^ The use of BAQLaVa to generate viral profiles based on metagenomic data takes advantage of novel algorithms as well as multiple integrated viral databases to improve viral detection and taxonomic classification.

A limitation of our study, consistent with observational studies generally, is in the ability to confidently ascertain causal effects of the virome on PD. Even with the inclusion of a prodromal group, it is still difficult, due to the observational nature of our study, to identify virome differences associated with the disease process versus those associated with reactions to changes in, for example, stool transit time. The relatively short time frame between stool collections (mean = 24.6 months) did not allow us to examine the relationship between the microbiome and “conversion” from prodromal to PD states. Our study would have benefited from adjustment for use of PD medications, such as Levodopa, which may influence the GV, but, unfortunately, PD medication data were not available in our cohorts. The impact of medication should be explored in future longitudinal studies on both the bacteriome and virome in PD. Although the classification accuracy of our RF model was modest (AUC of 0.63 for PD vs healthy controls and 0.67 for pPD vs controls), the result is consistent with a scenario in which the GV harbors a small but consistent signal for PD and pPD. The RF classification result appears to represent a population‐level shift in GV composition associated PD that is robust across the study sample, but not strong enough to accurately classify any given participant. Our RF results were also generally consistent with feature‐wise analyses in MaAsLin 2, showing that the study findings are generally robust to different quantitative modeling approaches.

In summary, this rigorously designed study nested within the NHS and HPFS, we identified 3 VGBs with elevated abundance and 1 with reduced abundance in PD, which were similarly related to pPD. The results of this study suggest that the endogenous human GV should be further explored as a potential biomarker of pPD and PD etiology, risk, and progression.

## Author Contributions

N.P., C.H., and A.A. contributed to the conception and design of the study; D.D., X.M., J.J., K.B., and M.S., contributed to the acquisition and analysis of data; D.D., X.M., J.J., and N.P. contributed to drafting the text or preparing the figures.

## Potential Conflicts of Interest

Nothing to report.

## Supporting information


**Supplementary Table S1.** Identified features in the VGBs associated with PD.

## Data Availability

Further information including the procedures to obtain and access data form the Nurses’ Health Study and Health Professionals Follow‐up Study is described at https://www.nurseshealthystudy.org/researchers (contact email: nhsaccess@channing.harvard.edu) and https://sites.sph.harvard.edu/hpfs/for-collaborators/. Metagenomic sequencing data and metadata for the study are available through dbGap (phs002193.v1.p1).
