# Supplementary material for: Shotgun Metagenomic Profiling of the Gut Virome in Prodromal and Confirmed Parkinson's Disease
Source: Ann Neurol. 2026 May 27;100(2):334–40. doi: 10.1002/ana.78243 (PMC13388000; doi:10.1002/ana.78243)
Supplement: Supplementary file 1 — Supplementary Table S1. Identified features in the VGBs associated with PD. [file ANA-100-334-s001.docx]

**Supplemental Table 1. Identified features in the VGBs associated with PD.**

| **VGB** | **Best Hit (% identity, alignment length)** | **Features** |
| --- | --- | --- |
| MVG041501 | BK037173.1 (*Caudoviricetes* sp)  97.7%, 22853 bp | Helicase, terminase, major & minor capsid proteins, six tail proteins |
| MVG081211 | BK046218.1 (*Caudoviricetes* sp)  74.4%, 4281 bp | Minor tail protein, portal |
| MVG081219 | BK046218.1 (*Caudoviricetes* sp)  74%, 4281 bp | Minor tail protein, portal |
| MVG098915 | BK020970.1(*Caudoviricetes* sp)  99.3%, 7350 bp | Major capsid protein, major head protein |
